# Cancer Related Fatigue and Quality of Life in Patients with Advanced Prostate Cancer Undergoing Chemotherapy

**DOI:** 10.1155/2016/3989286

**Published:** 2016-02-14

**Authors:** Andreas Charalambous, Christiana Kouta

**Affiliations:** ^1^Cyprus University of Technology, 15th Vragadinou Street, 3041 Limassol, Cyprus; ^2^University of Turku, 20014 Turku, Finland

## Abstract

Cancer related fatigue (CRF) is a common and debilitating symptom that can influence quality of life (QoL) in cancer patients. The increase in survival times stresses for a better understanding of how CRF affects patients' QoL. This was a cross-sectional descriptive study with 148 randomly recruited prostate cancer patients aiming to explore CRF and its impact on QoL. Assessments included the Cancer Fatigue Scale, EORTC QLQ-C30, and EORTC QLQ-PR25. Additionally, 15 in-depth structured interviews were performed. Quantitative data were analyzed with simple and multiple regression analysis and independent samples *t*-test. Qualitative data were analyzed with the use of thematic content analysis. The 66.9% of the patients experienced CRF with higher levels being recorded for the affective subscale. Statistically significant differences were found between the patients reporting CRF and lower levels of QoL (mean = 49.1) and those that did not report fatigue and had higher levels of QoL (mean = 72.1). The interviews emphasized CRF's profound impact on the patients' lives that was reflected on the following themes: “dependency on others,” “loss of power over decision making,” and “daily living disruption.” Cancer related fatigue is a significant problem for patients with advanced prostate cancer and one that affects their QoL in various ways.

## 1. Introduction

Prostate cancer is the most frequent malignancy among men, as it usually affects men over the age of fifty [1]. It is the most common cancer in men in North America and the second leading cause of cancer death among US men after lung cancer [2]. In Europe, prostate cancer is the third most common cancer site with 417,000 cases (12.1%) and the third most common cause of death from cancer in men with 92,000 deaths (9.5%) [3]. Prostate cancer itself and its treatment have been associated with side effects such as fatigue, insomnia, impaired sexual functioning, bowel problems, and urinary dysfunction that can have a negative influence on health-related quality of life [4]. Fatigue occurs both as a consequence of the cancer itself and as an adverse effect of cancer treatment [5]. Cancer related fatigue (CRF) can be defined as a persistent, subjective sense of tiredness related to cancer or cancer treatment that interferes with usual functioning [6]. CRF differs from “normal” fatigue that is caused by lack of sleep or tiredness in several ways; first, fatigue in cancer patients is more persistent, more devastating, and longer-lasting. Second, it involves physical, mental, and emotional fatigue, and third, it is not relieved by adequate sleep or rest [7]. The impact of CRF on the patient's ability to function is considerable; hence, this symptom is among the most distressing of all those reported by patients with prostate cancer [5] and one that leads to negative effects on self-care capacities and quality of life [8, 9].

Patients with advanced prostate cancer often undergo chemotherapy as a means to better control the disease when complete hormone refractoriness occurs [10] and to achieve improved survival rates. Chemotherapy often exacerbates the side effects of prostate cancer and negatively influences the QoL. Furthermore, a diminished QoL is often recorded in patients who develop biochemical failure that has been linked to fear of metastasis and disappointment from treatment failure [11, 12]. The effects on the QoL are complex and include the physical well-being of the patients, their mental well-being, role functioning, and levels of emotional distress [11]. Many prostate cancer patients may stumble upon late effects by the disease and the treatment including decreased physical function, increased body mass, impairment of bladder, bowel or sexual dysfunction, depression, and fatigue [9].

The increase in the survival time stresses that more attention is attributed to the improved understanding of the experienced impact of cancer and its treatment-related side effects on the patients overall QoL. CRF as a common and debilitating side effect with a complex etiology that can be attributed to cancer itself and its treatment and/or to a broad range of physical and psychologic comorbidities needs to be better understood as a means to improve its management and decreasing its negative impact on QoL [8, 11].

The aim of the study was to explore CRF that patients with advanced prostate cancer experience during their chemotherapy treatment as well as its impact on the overall QoL of these patients. The objectives set by this study were

1. to examine the incidence and severity of CRF in patients with prostate cancer undergoing chemotherapy,
2. to examine the nature of CRF in regard to the physical, affective, and/or cognitive dimensions,
3. to explore how CRF affects patients' everyday life.

## 2. Methods

### 2.1. Design, Sample, and Sampling

This was a cross-sectional descriptive study. Participants were randomly selected from the Out-Patients Oncology Clinics of two public hospitals in Cyprus. Eligible participants were those with an advanced prostate cancer diagnosis at least 6 months before the data collection; they received hormonal therapy with no response; they were on chemotherapy treatment with docetaxel as primary or combination chemotherapy (patients were recruited 2 days following the completion of the 3rd cycle of treatment), were able to speak and understand Greek, and had a score of >50 on the Karnofsky Performance Scale Index and a mean of >50 on the Attentional Function Index (AFI). Comorbidity data were obtained from medical reports using age-adjusted Charlson comorbidity index (a-CCI) [13]. CCI-aged adjusted score was computed, defining two comorbidity levels: ≤3 (low to moderate) and >3 (high). Participants were excluded from the study (a) if they were receiving or plan to receive conventional or complementary treatment for fatigue or (b) had an impaired cognitive ability or (c) were referred to palliative care services for end-of-life care. Additionally, 15 randomly selected patients (from the quantitative sample) were interviewed on the basis of the findings from the statistical analysis of the quantitative data in order to provide a deeper interpretation.

### 2.2. Instruments

Demographic information was obtained from the patients additionally to the Cancer Fatigue Scale (CFS) that assesses fatigue and EORTC QLQ-C30 and EORTC QLQ-PR25 that measures QoL. All necessary permissions were obtained by the developers.

### 2.3. Cancer Fatigue Scale

The CFS was used, which is composed of 3 factors and 15 items. It consists of three subjective dimensions: the physical, affective, and cognitive aspects of fatigue. Cancer fatigue scale is a brief and easy to use questionnaire that assesses the intensity of fatigue and the impact on a patient's daily life. There are three main advantages of this scale over other fatigue scales. First, this scale can be easily completed in a few minutes even by advanced cancer patients. Simplicity is an important advantage, because its main purpose is to assess the fatigue experienced by exhausted cancer patients. Second, this scale was specifically designed to reflect the nature of cancer related fatigue and to assess the physical, affective, and cognitive aspects of fatigue. Third, this scale showed good validity and reliability in a number of cancer patients sufficient for testing the psychometric properties of this 15-item scale [14]. The scale has English and German validated versions [15, 16]. The questionnaire was translated and culturally adapted in the Greek language based on the guidelines published by Beaton et al. [17]. The process of adapting the questionnaire proceeded through 5 phases. In phase one the questionnaire was forward translated in Greek by two bilingual translators, one of which was a healthcare professional and the other one an English literature professor. In phase two, the synthesis of the two translations occurred where a common consensus translation was produced. Following the synthesis of the common translation the instrument was back-translated to the original language by two translators (a healthcare professional and an English literature professor), totally blind to the original version. In the next phase an experts committee was formulated that consisted of health professionals (7), language professionals (2), and all the translators involved in the process (4). The committee produced a consensus version that corresponded to semantic, idiomatic, experiential, and conceptual equivalence. In the final phase, the questionnaire was pretested in a convenience sample of 15 patients. Following the completion of the questionnaire by the respondents they were asked to provide their perspective and understanding of each of the 15 items included in the instrument.

### 2.4. EORTC QLQ-C30

The European Organization for Research and Treatment of Cancer Quality Of Life Questionnaire (EORTC QLQ-C30) is an integrated system for assessing the health-related quality of life of cancer patients. It includes a total of 30 items and is composed of scales that evaluate physical (5 items) and emotional functioning (4 items), cognitive (2 items) and social functioning (2 items), and global health status (2 items). All of the scales and single-item measures range in score from 0 to 100. A high scale represents a higher response level. Thus a high score for a functional scale represents a high/healthy level of functioning; a high score for the global health status/QoL represents a high QoL, but a high score for a symptom scale/item represents a high level of symptomatology/problems [18]. The Greek valid version of the questionnaire was used in this study [19].

### 2.5. EORTC QLQ-PR25

The EORTC QLQ-PR25 is a 25-item questionnaire specifically designed for use among patients with localized and metastatic prostate cancer. It includes subscales assessing urinary symptoms (9 items), bowel symptoms (4 items), treatment-related symptoms (6 items), and sexual functioning (6 items) [18]. The questionnaire has been translated and validated in several languages including Greek [20].

### 2.6. Interview Guide

The qualitative data were collected with the use of an interview guide aiming to provide a deeper understanding on the ways that the patients perceived their lives being influenced by fatigue. In order to elicit this information patients were invited to provide their experiences in relation to the following open-ended question: “In what way(s) do you feel that your life was mostly affected by fatigue?” All interviews were digitally recorded and transcribed verbatim.

### 2.7. Ethical Considerations

The study conforms to all the principles described by the Helsinki declaration [21]. At every stage of the research, anonymity and confidentiality of the participants were preserved. The protocol of the study was reviewed by the Cyprus National Bioethics Committee (CNBC 2010/06) and was approved by the Ministry of Health of Cyprus (MH 5.04.019). Prior to the study, the participants were given a written letter informing them of the aims and objectives of the study, and written consent was obtained by every participant.

### 2.8. Data Analysis

Descriptive statistics were used for presenting the demographic data in addition to mean and standard deviation calculations for the scales used in the study. Independent samples *t*-test was used to examine the differences in the quality of life between the two groups, those who scored below the cut-off point of 18 and above the cut-off point of 18. Pearson correlation coefficients were computed between the CFS total/subscale scores and the EORTC QLQ-C30 fatigue subscale, as well as between the subscales of CFS and the corresponding EORTC QLQ-C30 functional scales (physical, cognitive). Regression analysis was performed, both simple and multiple regression, to examine the effect that CRF has on the QoL of patients with advanced prostate cancer. The dependent variable was the global health status (QoL) in all models and the independent variables were the total cancer related fatigue (CFS), as well as the subscales of CFS, when entered alone or simultaneously. A relation is considered statistically significant if *p* value < 0.05. We consider a relation marginally significant if the *p* value is smaller than 10%. Statistical analysis was performed using the IBM SPSS Statistics v.19 [IBM. Corp.]. The data deriving from the interviews were analyzed with the use of an inductive approach which is of thematic content analysis. Inductive approaches to data analysis involved analyzing data with little or no predetermined theory, structure, or framework [22].

## 3. Results (Quantitative)

### 3.1. Demographics

Out of the 216 eligible participants, 148 men (response rate 68.5%) diagnosed with advanced prostate cancer completed all the measurements and were included in the analysis. The largest proportion of men was aged between 61 and 70 years (32%) and came from Paphos (68%). Eighty-eight participants received their cancer diagnosis in the 6-month to 3-year period (59.5%) with the mean interval time of disease from the diagnosis of cancer until the completion of the questionnaire being 42.7 months. Patients in the group scoring above the cut-off had lower mean score compared to the group below the cut-off point (37.9 months versus 43.1 months). Based on the NCI Common Terminology Criteria for Adverse Events (NCI-CTCAE) the majority of the patients (81%) experienced mild to moderate anemia and 28 patients experienced Grade 3 anemia and were managed accordingly. The mean value on the Karnofsky Performance Scale was found 64 for the group scoring below the cut-off point and 57 for the group scoring above the cut-off point. The a-CCI score was calculated to be ≤3 in 97 patients (65.5%) and >3 in 51 patients (34.5%). Most men had a secondary school education (28%). Finally, most of these men had been supported both by their family and by cancer associations (90%).  Table 1 presents the demographics of the sample in detail.

### 3.2. Reliability

The reliability of the scales was measured with Cronbach's alpha, where values close to 1 show high internal consistency [23]. Cronbach's alpha for the CFS was 0.916, for the EORTC QLQ-C30 was 0.933, and for the QLQ-PR25 was 0.896.

### 3.3. Cancer Related Fatigue

The results showed that the values of the CFS total scale ranged from 5 to 52, with a mean of 26.77, which is below average and could be interpreted as a low to moderate level of fatigue. The “physical” subscale ranged from 0 to 25, with a mean of 11.37, which is below the average and could also be interpreted as low to moderate level. The results showed that 64.2% scored below 14 on the physical subscale. The “affective” subscale ranged from 2 to 16, with a mean of 10.38, which is actually above the average. This showed that respondents had on average a rather high level of fatigue related to affective issues that included energy, concentration, encouragement, and interest in things, compared to other types of fatigue. In fact, the results showed that 64.2% of respondents had a level of affective fatigue above 8. The “cognitive” subscale ranged from 0 to 14, with a mean of 5.02, which is below the average, showing a low level of cognitive-related fatigue. As high as 77.7% of respondents had a low (up to 8) level of cognitive-related fatigue.

To examine the incidence and severity of CRF the score of 18 was used as the cut-off point. This decision was informed by the results of previous studies where a score of 18 was consistently found as the cut-off point for screening CRF [14, 24]. The results showed that 49 patients or 33.1% had a score up to 18 and 99 patients or 66.9% had a score above the cut-off point of 18. In other words, a very high percentage of patients experienced fatigue (total fatigue scale) in this sample.

### 3.4. Quality of Life (QLQ-C30 and QLQ-PR25)

The results showed that the functional scales were on a good level (reported by high values) showing no serious problems on these functions (Table 2). The function with the highest score was the social function (76.4 ± 20.9), followed by cognitive functioning (71.4 ± 20.8). However, patient appeared to experience diminished emotional functioning, a fact that was reflected in the recorded rather low level (48.2 ± 26.5). The symptom scales were also at satisfactory levels (reported by low values). The best results regarding the mean levels of symptoms were for the diarrhea and constipation scales (23.2 and 24.1, resp.); however they both had very high standard deviations indicating high variability in the respondents' answers. The symptom that the patients perceived to experience the most problems with was fatigue (51.6 ± 30.9). This finding corresponds to the high percentage of participants found to report fatigue through the CFS.

The results showed that for the urinary, bowel, or treatment-related functions the level of symptoms is below average (2.5 as the middle value), showing that the patients did not have severe problems with regard to these functions. Additional results have indicated that for the urinary scale 67.6% of the patients scored below 2.5, while for the bowel and treatment-related scales all respondents scored below 2.5. The scale of sexual functioning had a high mean, indicating a high level of problems in this area, with the maximum reaching the possible maximum of 5. In total 91.2% of the respondents scored above 2.5 on this scale.

### 3.5. The Effect of Cancer Related Fatigue on Patients' Quality of Life

A *t*-test was performed to examine if there were differences in the QoL between the two groups (less than 18, more than 18). The results showed that there are significant differences in the QoL between patients that experienced CRF and those who did not (*p* < 0.001). The mean levels of QoL of those that scored below the cut-off point were much higher (72.1) compared to those that scored above the cut-off point of 18 (49.1). Therefore, CRF appears to have a negative impact on the levels of QoL.

The results from regression analysis (Table 3) showed that CRF has a negative effect on the QoL of patients with prostate cancer. Explicitly, the total CRF scale had a negative coefficient (−0.943) and a *p* value < 5%, which indicates that the higher the levels of overall fatigue, the lower the levels of QoL. The model *R* square is equal to 0.357, showing a rather satisfactory fit. When each of the subscales of the CFS was entered independently they all had a statistically significant negative effect on the QoL, with negative coefficients and *p* values < 5%. Interestingly enough, when the three subscales were entered simultaneously, only the two subscales physical and affective had a significant effect on QoL (with *p* values < 5%), while the third subscale, related to cognitive fatigue, was not significant anymore (*p* value = 0.237). This shows that the first two scales have a more significant effect on the QoL, compared to the cognitive scale. The effect of cognitive functioning disappears when it is combined with the other functioning scales. This could also be seen from the very low *R* square (0.063) when the cognitive functioning was included by itself, in model 4. The very low *R* square shows that this variable is not sufficient enough to explain the variation in “quality of life,” and other variables should be considered for the model.

Separate analyses were also performed for patients with CFS below 18 and those above 18, for comparison reasons. The results from the separate analyses of patients with low (Table 4) and high levels of fatigue (Table 5) showed that the QoL of the two groups of patients is affected by the total fatigue scale (although, for the lower fatigue group, CFS affects the patients in a less significant way). QoL appeared to be affected by different fatigue dimensions: for patients with high levels of fatigue, the QoL is affected only by the affective subscale. In patients with low levels of fatigue, the QoL is affected by both the physical and the affective subscales.

Pearson correlation coefficients were found significant between the CFS total/subscale scores and the EORTC QLQ-C30 fatigue subscale, as well as between the subscales of CFS and the corresponding EORTC QLQ-C 30 functional scales (physical, cognitive) (Table 6). The most important correlation was between CFS and EORTC-fatigue since the two fatigue scales were indeed positively and significantly correlated. The correlations between the subscales of CFS and EORTC-fatigue were also significant. The correlations between the CFS-physical and EORTC-physical subscales and between the CFS-cognitive and EORTC-cognitive subscales were found significant (*r* = −0.732 and *r* = −0.500, resp.). The negative signs were expected, due to the scoring of the EORTC functional scales, where high values show good functioning of the patient as opposed to the CFS subscales, where high values show high levels of fatigue.

## 4. Results (Qualitative)

Following the principles of data saturation, 15 patients diagnosed with advanced prostate cancer were randomly selected from the sample of 148 patients to be interviewed. The researchers considered that data saturation was reached when there was enough information to replicate the study [25], when the ability to obtain additional new information has been attained [26], and when further coding was no longer feasible [26].

The main themes for each core aspect are presented as these were described by the patients. These themes were based on their interviews and illustrated by verbatim quotes from participant interviews.

Although the Cancer Fatigue Scales including the CFS provided some information about the ways the patient's life can be affected by fatigue, these are rather general descriptions failing to explicitly describe the problem(s) that the patients experience in everyday life. The qualitative analysis revealed the following main themes that reflected on the ways that the patients perceived their lives being influenced by fatigue: “dependency on others,” “loss of power over decision making,” and “daily living disruption.” The interviews revealed that fatigue had a great impact on patients' lives by preventing them from leading a normal life and conducting their daily routine.

*Being unable to care for yourself is the first thing that comes to my mind. Prior to treatment and the onset of this symptom I was able to care for myself without any problems or help from others. Now I find it extremely difficult and burdensome to do the same things of everyday life…imagine that even having a shower is an activity that causes me stress as it means pushing my body to the limit in order to accomplish this…I never thought that it would have been an “accomplishment” simply to have a shower.*

Under the same theme, the patients also revealed that being fatigued limited some of their social activities. The activities that were mostly influenced by fatigue were patients' visits to friends and relatives and participation in other social events such as going out to restaurants. More socially active patients seemed to be affected more by the social impacts of fatigue as they viewed the whole situation as “being imprisoned.”

*I used to enjoy the walks in the park, particularly on the weekends, it was a kind of way out from the disease and the treatment, even for a while, and a chance to enjoy nature and get together with some friends, it was definitely one of the few spices in my life, but I feel that this was taken from me by this constant body and mind tiredness.*

*There are things that you take for granted before you get sick and things that you miss when you are sick. It sounds odd, but since all these started (means chemotherapy and fatigue) I have been avoiding my friends…we used to do so many things together…but now even the simplest ones cause me stress such as going out for a nice meal…I don't want my friends to see me like this…it's the forgetting, the difficulty in speaking and of course the physical exhaustion that scare me the most and the ones that I want to keep for myself.*

*It feels like I was sentenced to home imprisonment by this situation. Nothing that involves me going out is simple anymore. I can't walk far, I get tired with minimum effort and the worst of all is that I can't get myself out of the bed these days. The home provides me safety but at the same time isolates me from the rest of the world….*

The theme “daily living disruption” was closely related to the theme “dependency on others” as the patients saw their limited ability to perform their activities of daily living as the reason for their increased dependency on others.

*It is hard for me to answer this question. What is the worst, not been able to continue living as usual or being partially or totally depended on my wife and children? When cancer presented itself in my life I slowly saw my independence fading. Fatigue was the final stroke…that took this (independence) away.*

In the interviews the patients that experienced fatigue appeared to perceive their increased loss of independence as a source of burden for their caregivers. This role was often assumed by one member of their family (usually the spouse or children) that caused additional stress to patients. This perceived stress was due to the fact that the caregivers needed to spent significant time with the patients, often at the expense of their own families and work.

*The feeling is terrible, relying on others to do the things I used to do; I have become somewhat a “burden” to them. I often see that they need to make sacrifices to keep me happy and often these are huge as it often means less time with their own families, I am not forgiving myself for this. I know my family loves me and they are willing to help in any way they can, but it is the psychological impact of this that I cannot come to terms with.*

*The problem (referring to fatigue) increasingly got worse, meaning that my daughter needs to spend more time with me, last week she had to take 2 days off work just to be able to care for me in the mornings…I felt it was so unfair to her, putting her job at risk just to be with me when I needed her the most…I feel trapped in an awful situation with no options.*

The theme “loss of power over decision making” was also related to the theme “dependency on others” as the patients in the interviews commented on the fact that they were relying on others to decide on their behalf on what and when to do most of the things of their daily living.

*[…] Nothing is the same anymore, I lost my appetite to do anything nowadays, and how can you be happy if you cannot do the things that bring you pleasure at the time and place that you want? It feels like that I am living the life of others…I get to do the things that others are in the mood for and what others decide on my behalf. I am sorry for myself because I do not see the “living” in my life anymore.*

The theme “loss of power over decision making” also consisted of the situations described by the patients where they felt unable to make decisions regarding their selves. Experiences were described such as patients willing to go out, play with their grandchildren, or even get out of the bed but due to the fatigue they were experiencing they felt powerless to carry out these decisions.

*The last few days have been terrible. I couldn't even get myself out of the bed the matter how hard I tried. Deciding to do something is one thing but the power to make that thing happen is another? The spirit is willing but the flesh is weak…It is times such as these that I say to myself that deciding for anything has become a joke.*

*Last week my grandchildren came for a visit, they hadn't seen me for a month or so due to the treatment I was taking, the kids wanted me to play with them as we used to do, well I decided that I should do this, it is expected of me regardless of anything else, so I found the energy to engage in the play with them, but within minutes my powers gave up, I was so frustrated with myself and the kids were so disappointed in me…I could see it in their eyes.*

## 5. Discussion

This study explored the topic of CRF and its subsequent impact on QoL in patients diagnosed with advanced prostate cancer during the period of active treatment with chemotherapy. Explicitly the study shed light on the incidence, severity, and nature of cancer related fatigue experienced in this group of patients.

The results demonstrated that fatigue is a rather common symptom among patients with advance prostate cancer undergoing treatment. This was supported by the high levels of fatigue reported in this sample with almost two-thirds of the patients (66.9%) reporting fatigue as a problem. This is also consistent with other studies [27, 28] indicating that fatigue is gaining more access by researchers around the world acknowledging CRF as one of the most significant and long-term consequences of cancer and its treatment in this group of patients. The remaining 33.1% of the sample did not appear to have clinical fatigue, a finding that is not uncommon in the relevant literature [27, 29]. This result should be interpreted in the light that the patients who did not appear to have clinical fatigue had a better mean score on the Karnofsky Performance Scale indicating a lesser burden of the disease and a higher mean interval time of disease from the diagnosis of cancer until the completion of the questionnaire. Another possible explanation is that these patients could have been used to feeling fatigued and had already reduced their standards of fatigue to lower levels. Another possible explanation is that these patients may have found effective coping strategies that helped them manage fatigue in their daily living.

Cancer related fatigue is a complex construct comprising of different consisting elements. Bower [7] argued that CRF consists of physical, mental, and emotional elements. Similarly Okuyama et al. [15] acknowledged the complexity of encapsulating CRF and proposed the consideration of physical, affective, and cognitive issues in the process of assessing the construct. The varying nature of CRF experienced by the patients in this study was also recorded in the findings. Therefore, the results showed that different patients acknowledged different problems (physical, affective, and cognitive) in relation to their CRF. The affective problems that include issues such as energy, concentration, and interest in things however appeared to pose the greatest challenge for the patients in this study, an aspect that was reflected in the high levels of affective fatigue recorded in the 64.2% of the patients. All three subscales of CRF were found to influence the patients QoL. However, the results demonstrated that problems related to physical functioning (feeling tired, exhausted, reluctant, and fed up) and affective functioning (e.g., lacking interest in anything, energy, encouragement, or concentration) appeared to severely affect the QoL of a patient with advanced prostate cancer undergoing chemotherapy. Patients who only experienced cognitive problems (e.g., making errors while speaking, becoming forgetful) were found to have slightly diminished QoL, and not to the same extent as those who also reported physical or affective functioning problems. Okuyama et al. [14] in a sample with thirty-seven consecutive patients ambulatory patients diagnosed with advanced lung cancer found that one-third of the patients reported that fatigue was related to physical issues while about one-fifth of patients reported that fatigue was related to affective issues.

It is well established in the relevant literature that cancer therapies such as chemotherapy are accompanied by side effects such as fatigue, pain, and reduced physical functioning and QoL [30]. In this study, fatigue is reported by a mean number of patients as a problem that negatively affects their QoL, a finding that coincides with the findings of preceding studies [14, 31–33]. Sternberg et al. [34] in a study with 797 patients with metastatic castration-resistant prostate cancer after docetaxel chemotherapy identified fatigue as the main side effect of chemotherapy that negatively influenced the patient's QoL. However, few studies in the literature provided evidence that CRF is not correlated with QoL. For example, Dash et al. [9] in a study with 40 patients that were treated and followed up with hypofractionated stereotactic body radiation therapy-SBRT for clinically localized prostate adenocarcinoma explored the incidence of fatigue and its association to QoL. They found that fatigue was not a major side effect for this group of prostate cancer patients and no correlation was found between CRF and QoL.

Despite the evidence showing that a majority of patients will experience some level of fatigue during their course of treatment [35] and approximately 30% of patients will endure persistent fatigue for a number of years after treatment [36], CRF was poorly addressed within the clinical setting. This was the result on focusing on other symptoms such as pain, nausea, and vomiting and because fatigue was considered an unavoidable and not life-threatening symptom to be endured rather than treated [37]. The interest in these symptoms is largely determined by the assumption that they can have a negative effect on the patient's overall QoL. Therefore this study has provided evidence on the possible association between CRF and patient's QoL. The results showed that when correlating the QoL with the CFS for both groups, under and over the cut-off point of 18, the mean of the group below 18 was much higher than the group above 18, suggesting that CRF does affect QoL.

Fatigue is adversely associated with activity level and functional capacity; a consistent decrease in the amount of daily activity over the often lengthy period of cancer treatment can eventually lead to a reduced tolerance for normal activity and, hence, high levels of fatigue and low levels of QoL [38]. A study by Braun's et al. [2] showed that among the QLQ-C30 functioning scales the highest (best) mean score (83.4) was recorded for physical functioning. The quantitative results of the current study showed that the physical function mean score was 62.25, indicating that even though their physical functioning was affected by fatigue, the patients did not experience serious issues regarding this function. Reflecting on the qualitative data, one realizes that for the patients that experienced fatigue the limited physical functioning influenced their ability to continue living as usual and perform the activities of daily living. These findings coincide with those of preceding studies. For example, Charalabopoulos et al. [39] support that there is a range of physical difficulties that may be experienced by men with advanced prostate cancer which can affect their QoL. Similarly in Curt et al. [8] in a survey of 379 cancer patients having a prior history of chemotherapy, patients reported low physical status; they commonly described a significantly diminished energy level, a need to slow down from a normal pace, and a general sense of sluggishness or tiredness and perceived fatigue as the obstacle to live a “normal” life. These issues were correlated to low QoL levels. Furthermore, Morrow [40] states that fatigue has a strong and direct impact on all aspects of the QoL of patients with cancer, particularly their physical well-being.

The results in this study showed that the sexual function of the patients was affected, while the bowel and urinary functions were affected to a lesser degree by the CRF. Numerous studies have also revealed the impact of prostate cancer treatment on sexual, urine, and bowel function [41–43]. Talcott et al. [44] in their study also reported that cancer treatment affects sexual function. In Maliski et al. [45] in a longitudinal prostate cancer QoL study with 402 men, a 10% of the total number of patients reported sexual dysfunction which was correlated with reported fatigue.

While other studies [27, 39] found that CRF affects patients' ability to perform daily activities and limits their personal and social role functioning, in the present study, all functional scales were on a good level (above average) with the best function being the social function. However, the qualitative data analyses revealed that the impact of fatigue on the person's social activities remains a problem for the patients experiencing fatigue. Based on the patients' experiences fatigue prevented them from performing some of their usual social activities such as visiting friends or going out. Although their social functioning may well be high (as presented by the quantitative data), their ability to perform the activities that support social functioning is usually negatively affected. A possible explanation for the high levels of social functioning reported is the level of support that patients received during the treatment. The support provided to the patients by family, friends, and the cancer patients associations during treatment balanced the loss of usual social interactions and therefore any decreased social interaction was not perceived. A percentage of 89.9% of the patients reported that they were actively supported by their family as well as the cancer patients associations. The Cyprus family has through history benefited from strong relationships that become even more dynamic when a life-threatening disease affects a family member. For Cypriots cancer possesses a social disease and the response is collective by the other members of the family who are supportive, affective, and loving to the ill person strengthening his or her support system [46]. This is also noted in the work of Olson et al. [47] who also provided another possible explanation to this finding. They argue that the individuals receiving chemotherapy had already limited social interactions due to risk of infection, and thus, they did not link decreased social interaction to fatigue. The qualitative data in the current study provided evidence that the provision of social support by the family comes with a high cost for the person who assumes the role of the caregiver. Based on the patients' descriptions often their family caregivers are forced to take time off from their work in order to support them while the time spent with their own families is reduced for the same reason. Other studies also state that support is a countermeasure for cancer patients. For example, Talcott [44] comments that social support improves patient outcomes in prostate cancer. Bower [7] also supports that any kind of support may have beneficial effects on patient's fatigue. In Ahlberg et al. [38] study, the participants in the group that received some support showed reduced symptoms of fatigue and pain, and social role function was significantly higher than that of the patients in the control group. In addition, in Curt et al. [8] study where patients had a low social functioning, they also reported a low financial status. The findings by Curt et al. [8] provide a possible explanation for the high level of social function reported in the current study since the scale of financial problems was in satisfactory levels.

The functional scale which had a rather low level was emotional functioning. A possible explanation for these findings is that when fatigue is in relatively high levels, emotional functioning is decreased and vice versa. This finding contradicts the fact that the patients were receiving support by the family or/and the cancer patient associations. An explanation is that the type of support provided by these support groups did not attribute adequate attention to emotional aspects. This is a consistent finding in the relevant literature. For example, Yucel et al. [48] in a study with 367 cancer patients who received curative radiotherapy found that fatigue negatively influenced the emotional functioning of the patients and subsequently their perceived levels of QoL. Similarly, Kawaguchi et al. [49] analyzed 93 postoperative patients with breast cancer before pharmacist counseling for adjuvant systemic therapy. Their results indicated that fatigue and emotional functioning were strong factors affecting QoL. Fatigue may be particularly upsetting to men who have led active and independent lives. Some men often consider themselves as the protectors and providers of their family; since cancer and its treatment, along with CRF, are considered as limitations to patients' physical functioning, one can claim that their emotional status will be affected as well [49]. Morrow [40] agrees that patients experiencing fatigue must frequently engage in unwanted behaviors, such as lying down or taking naps, in an attempt to cope with their fatigue; this change in daily activity and self-sufficiency may be demoralizing and discouraging. The qualitative findings also support this claim. Patients appeared to be frustrated by their limited input in decision making regarding their own living in terms of performing the activities of daily living and engaging in social activities that provided them with the sense of happiness. They appeared disappointed in failing to meet the expectations of others due to their physical impairment. Another probable explanation may be that although most of the patients had some kind of support in coping with cancer and its treatment and had a positive performance status, they probably were disturbed by the disease itself or the treatment [31]. Okuyama et al. [14] and Hofman et al. [5] found similar results, suggesting that the impact of fatigue on daily activities may extend in their emotional functioning.

Another finding of this study is that measures of cognitive performance did not associate strongly with fatigue. This finding was also supported in Green et al. [50] and Mallinson et al. [32]. A reason for this may be that cognition may not be significantly impaired in patients without central nervous system disease at treatment onset [51]. This finding contrasted with other studies [38] who reported negative correlations between fatigue and cognitive performance.

This study was limited by the somewhat small sample size. This was attributed to the relatively small number of patients with advanced prostate cancer that undergo chemotherapy. However, the fact that patients were randomly selected from the only 2 public hospitals providing such care to prostate cancer patients increased the generalizability of the findings. In the literature a cut-off point for the subscales of the CFS was not advocated; therefore the three subgroups (physical, cognitive, and affective) could not be examined thoroughly in this study.

## 6. Conclusions

Authors conclude that fatigue is one of the most critical problems for patients with advanced prostate cancer in the period of receiving chemotherapy. This study has revealed that patients experience CRF differently and therefore a deeper assessment is needed to identify whether fatigue is related to physical, affective, or cognitive issues. Fatigue is a subjective experience and therefore it should be systematically assessed using patients self-reports and other sources of data such as individual interviews. Because of functional insufficiencies patients with advanced prostate cancer suffer from persistent emotional and social distress and a reduced QoL. Healthcare providers need to focus on emotional functioning as it was found to be negatively associated with CRF. Routine screening for fatigue is essential to assess patients throughout the cancer trajectory. It is hoped that these findings will contribute to the better understanding of cancer related fatigue in this population and facilitate the need for recognition, evaluation, monitoring, and documentation and prompt treatment in this group of patients.

## Figures and Tables

**Table 1:** Demographics (*N* = 148).

Variable	*N*	%
(1) Area of residence		
Nicosia	34	23.0
Limassol	7	4.7
Paphos	101	68.2
Larnaca	6	4.1
(2) Age		
40–50	25	16.9
51–60	37	25.0
61–70	47	31.8
>70	39	26.4
(3) Time from diagnosis		
6 months–3 years	88	59.5
4–6 years	45	30.4
7–10 years	6	4.1
>10 years	9	6.1
(4) Level of education		
No formal education	27	18.2
Primary school	37	25.0
Secondary school	42	28.4
Higher education (college/polytechnic)	21	14.2
University degree	21	14.2
(5) Supporting system		
Family (spouse, children)	10	6.8
Cancer patient association	5	3.4
Family and cancer association	133	89.9

**Table 2:** Descriptive statistics for EORTC QLQ-C30 (*N* = 148).

Scale	Mean	Standard deviation
QLQ-C30		
Global health status/QoL	56.70	19.01
Functional		
Physical function	62.25	26.97
Role function	55.07	32.22
Emotional function	48.25	26.55
Cognitive function	71.40	20.76
Social function	76.35	20.85

Symptoms		
Fatigue	51.58	30.89
Nausea/vomiting	37.95	34.55
Pain	43.02	30.62
Dyspnoea	34.91	23.11
Insomnia	36.26	33.43
Appetite loss	40.54	30.75
Constipation	24.10	30.32
Diarrhoea	23.20	39.20
Financial problems	36.04	24.44

**Table 3:** Regression analysis models for the effect of cancer related fatigue on quality of life (*N* = 148).

Model	Variables	Beta	Standard error	*p* value	*R* square
1	CFS	−0.943	0.105	<0.001^*∗∗*^	0.357

2	Physical	−1.466	0.180	<0.001^*∗∗*^	0.312

3	Affective	−2.559	0.319	<0.001^*∗∗*^	0.306

4	Cognitive	−1.361	0.433	0.002^*∗∗*^	0.063

5	Physical	−1.190	0.234	<0.001^*∗∗*^	0.432
	Affective	−1.697	0.328	<0.001^*∗∗*^	
	Cognitive	0.521	0.438	0.237	

^*∗∗*^Statistically significant at the 1% level.

**Table 4:** Regression analysis models for the effect of cancer related fatigue on quality of life: patients with CFS below 18 (*N* = 49).

Model	Variables	Beta	Standard error	*p* value	*R* square
1	CFS	−1.275	0.496	0.013^*∗*^	0.123

2	Physical	−1.575	0.679	0.025^*∗*^	0.290
	Affective	−1.056	0.473	0.031^*∗*^	
	Cognitive	1.724	1.039	0.104	

Statistically significant at the 1% level. ^*∗*^Statistically significant at the 5% level.

**Table 5:** Regression analysis models for the effect of cancer related fatigue on quality of life: patients with CFS above 18 (*N* = 99).

Model	Variables	Beta	Standard error	*p* value	*R* square
1	CFS	−0.558	0.203	0.007^*∗∗*^	0.072

2	Physical	−0.545	0.336	0.108	0.191
	Affective	−2.236	0.694	0.002^*∗∗*^	
	Cognitive	0.713	0.533	0.184	

^*∗∗*^Statistically significant at the 1% level. Statistically significant at the 5% level.

**Table 6:** Pearson correlation coefficients of CFS with EORTC QLQ-C30.

	CFS-physical	CFS-affective	CFS-cognitive	EORTC-physical function	EORTC-cognitive function	EORTC-fatigue
CFS	0.935^*∗∗*^	0.663^*∗∗*^	0.726^*∗∗*^	−0.675^*∗∗*^	−0.550^*∗∗*^	0.745^*∗∗*^
CFS-physical		0.449^*∗∗*^	0.621^*∗∗*^	−0.732^*∗∗*^	−0.586^*∗∗*^	0.817^*∗∗*^
CFS-affective			0.180^*∗*^	−0.328^*∗∗*^	−0.153	0.345^*∗∗*^
CFS-cognitive				−0.422^*∗∗*^	−0.500^*∗∗*^	0.469^*∗∗*^
EORTC-physical function					0.632^*∗∗*^	−0.767^*∗∗*^
EORTC-cognitive function						−0.666^*∗∗*^

^*∗*^Correlation is significant at the 5% level. ^*∗∗*^Correlation is significant at the 1% level.
